# Graves' Disease Presenting as a Unilateral Breast Mass

**DOI:** 10.1155/2022/6641661

**Published:** 2022-12-22

**Authors:** Neeka N. Akhavan, Edlira Maska

**Affiliations:** Department of Internal Medicine, University of Florida, Gainesville, Florida, USA

## Abstract

**Introduction:**

Graves' disease is an autoimmune thyroid disorder that is the most common cause of hyperthyroidism. Common manifestations of Graves' disease include weight loss, palpitations, heat intolerance, fatigue, tremors, and exophthalmos, occurring in more than 50% of patients. In rare cases, findings may predominate in one organ system; isolated findings of diarrhea, anxiety, or gynecomastia (as in our case) may occur, distracting from the correct diagnosis. *Case Report*. We report on a 37-year-old male who presented to the primary care clinic with the chief complaint of a tender right-sided breast mass and with an associated loss of appetite and forty-pound weight loss. Laboratory evaluation revealed suppressed TSH and elevated free T4. A bilateral diagnostic mammogram revealed bilateral gynecomastia. A nuclear medicine thyroid uptake scan was subsequently ordered, which showed the diffusely enlarged thyroid gland with homogenous increased uptake throughout, consistent with Graves' disease.

**Conclusion:**

It is important to keep a high index of suspicion for thyroid disease as hyperthyroid states may be deceiving in presenting with single organ system involvement. Although it is rare, gynecomastia is a known finding in Graves' disease and can be the presenting sign. Patients with unexplained gynecomastia or breast masses should be screened for thyrotoxicosis.

## 1. Introduction

Graves' disease is an autoimmune thyroid disorder that is the most common cause of hyperthyroidism. The disease is characterized by a hyperthyroid state secondary to autoantibodies that bind to and activate thyrotropin receptors, leading to an increase in thyroid hormone production [1]. Patients of any age can be affected, however, the peak incidence is reported between the ages of 30 and 50 [2]. Common manifestations of Graves' disease include weight loss, palpitations, heat intolerance, fatigue, tremors, and exophthalmos, occurring in more than 50% of patients [3]. Rare manifestations such as localized dermatopathy and thyroid acropachy (i.e., clubbing) have been reported in less than 1% of cases. Men may present with decreased libido or erectile dysfunction, and women may present with irregular menses [1]. In rare cases, findings may predominate in one organ system; isolated findings of diarrhea, anxiety, or gynecomastia (as in our case) may occur, distracting from the correct diagnosis. Gynecomastia is a common finding in hyperthyroidism, but it is rarely the presenting symptom [4]. We present a case of Graves' disease with a less common presentation of a unilateral breast mass.

## 2. Case Report

A 37-year-old Caucasian male presented to the primary care clinic with the chief complaint of a tender right-sided breast mass. The patient also reported decreased appetite and a forty-pound weight loss over the preceding five months. The breast mass had been present for about six weeks and was not associated with galactorrhea. The patient denied any fevers or night sweats and denied taking any hormonal or nutritional supplements. There was no reported family history of breast cancer or endocrine disorder. During a physical examination, vital signs were within normal limits, and the patient was noted to be thin, with a BMI of 21.6. Breast examination revealed a one-centimeter-by-one-centimeter, tender, right-sided mass without nipple discharge. The thyroid gland was nonpalpable, and there was no evidence of lymphadenopathy.

**Figure 1:**
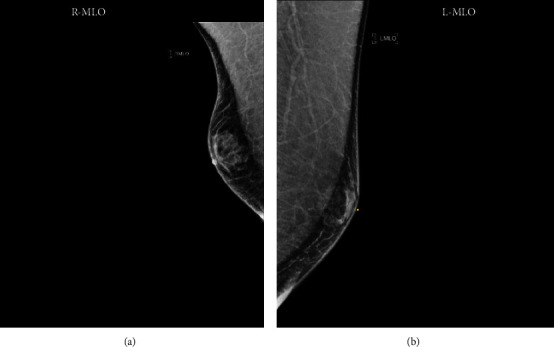
Magnetic resonance imaging (MRI) of the (a) right breast and (b) left breast showing flame-shaped subareolar densities consistent with gynecomastia.

Laboratory evaluation revealed TSH of 0.01 mIU/L (reference range 0.40–4.5 mIU/L) with free T4 3.3 ng/dL (reference 0.93–1.7 ng/dL) and total T3 335 ng/dL (reference 80–200 ng/dL). The thyroid-stimulating immunoglobulin level was noted to be >500 (normal <122%). Prolactin was found to be 7.2 ng/mL (reference range 4.0–15.2 ng/mL), and testosterone was noted to be 624 ng/dL (reference range 800–1080 ng/dL). A bilateral diagnostic mammogram revealed bilateral gynecomastia, right greater than left (Figure 1). A nuclear medicine thyroid uptake scan was subsequently ordered, which showed the diffusely enlarged thyroid gland with homogenous increased uptake throughout, consistent with Graves' disease.

On initial presentation, there was a high index of suspicion for breast cancer due to presenting symptoms of the right-sided breast mass associated with significant weight loss. The bilateral diagnostic mammogram and ultrasound were ordered for further workup of possible malignancy. Hyperthyroidism was also considered due to weight loss and palpitations; however, it was initially lower on differential diagnoses.

The patient was started on atenolol 25 mg daily upon laboratory diagnosis of hyperthyroidism. After confirmation of Graves' disease on the thyroid uptake scan, the patient was referred to endocrinology. He was subsequently prescribed methimazole 10 mg twice daily.

After two months of treatment with twice daily methimazole, repeat bloodwork showed TSH 0.45 mIU/L (within normal limits), free T4 0.39 ng/dL (normal 0.93–1.7 ng/dL), and total T3 58 (normal 80–200). On repeat evaluation, the breast mass was no longer palpable, and the patient reported significantly improved energy levels and decreased palpitations. The weight of the patient, however, was unchanged. Over the last five years, multiple attempts at tapering off methimazole have been unsuccessful. He has been maintained on 5 mg methimazole daily with stable thyroid function labs. He has declined definitive therapy with radioactive iodine ablation.

## 3. Discussion

Gynecomastia is defined as an enlargement in glandular tissue of the breast, which may present as unilateral or bilateral breast enlargement. This condition is commonly seen in patients with chronic liver disease and as a result of certain drugs [5]. Although it is rare, gynecomastia has been described as a common finding in hyperthyroid states, specifically in Graves' disease [6]. As a cutaneous feature of thyrotoxicosis, gynecomastia can be seen in 10–40% of cases; however, it is rarely seen as the presenting feature of the disease. It has been reported as a presenting feature of hyperthyroidism in less than ten cases to date [7].

The development of gynecomastia involves an imbalance between free estrogen and free androgen actions in breast tissue. Breast tissue enlargement occurs when there is a relative increase in estrogen production. In hyperthyroid states, the increase in concentration of sex hormone-binding globulin binds testosterone more avidly than estrogen, leading to an increase in the concentration of bioavailable estrogen [6]. Treatment of hyperthyroidism with control of thyrotoxicosis results in resolution of gynecomastia [7].

Our patient presented with the chief complaint of a unilateral breast mass associated with forty-pound weight loss. This presentation was initially concerning to the patient and physician for breast cancer due to the predominant involvement of only one organ system. On further investigation with diagnostic mammogram, the patient was found to have bilateral gynecomastia. Laboratory work and thyroid uptake scan confirmed diagnosis of Graves' disease.

It is important to keep a high index of suspicion for thyroid disease as hyperthyroid states may be deceiving in presenting with single organ system involvement. Although it is rare, gynecomastia is a known finding in Graves' disease and can be the presenting sign. Patients with unexplained gynecomastia or breast masses should be screened for thyrotoxicosis [8].

## Data Availability

No data were used to support the findings of this study.
